# Can social media be a threat or an opportunity to public health via the impacts on diet quality?

**DOI:** 10.3389/fpubh.2025.1679178

**Published:** 2025-12-10

**Authors:** Busra Ayhan, Suleyman Kose, Meryem Saban Guler, Saniye Bilici

**Affiliations:** 1 Department of Nutrition and Dietetics, Faculty of Health Sciences, Gazi University, Ankara, Türkiye; 2 Department of Nutrition and Dietetics, Faculty of Health Sciences, Artvin Çoruh University, Artvin, Türkiye

**Keywords:** diet, nutrition, adolescent, public health, quality of life

## Abstract

**Background:**

Previous studies have emphasized the influence of social media on people’s eating habits and preferences. Studies have indicated that social media can influence diet quality through a variety of processes in both positive and negative ways. But, it is still unknown how social media’s widespread use in daily life affects users’ nutritional condition.

**Objective:**

A survey form covering the frequency of social media use (SMU), topics related to nutrition followed, and accounts followed was administered by researchers. Participants self-reported age, weight, and height and provided 24-h dietary recall records.

**Methods:**

A total of 1,241 university students, all with at least one social media account, participated in the study. A survey form covering the frequency of SMU, topics related to nutrition, and accounts followed was administered by researchers. Participants self-reported age, weight, and height, and provided 24-h dietary recall records. Nutrient Adequacy Ratio (NAR) and Mean Adequacy Ratio (MAR) scores were calculated based on the 24-h dietary recall records to assess diet quality.

**Results:**

Among the persons who use social media frequently, 39.4% were classified as male, while 60.6% were classified as female. The study also found that 48.7% of students who use social media rarely and 41.0% frequently are enrolled in health-related departments. Instagram was the most popular platform among students and the source of healthy nutrition recipes. Accordingly, it is seen that the frequency of social media use does not give a statistically significant result with BMI (*p* < 0.05). Students in health-related departments had significantly reduced energy intake compared to other departments (*p* < 0.05). However, they exhibited elevated consumption of fiber and vitamin A. Energy, protein, and carbohydrate intakes were not significantly different between students who used social media rarely and frequently. Calcium and potassium levels of those studying in the health department who use social media frequently were found to be significantly higher than those who use social media rarely (*p* < 0.05). Participant MAR scores and classifications were not significantly different by academic department or SMU classification (*p* > 0.05).

**Conclusion:**

Social media holds potential as a tool for promoting healthy food choices among university students who actively engage with it.

## 1 Introduction

Worldwide, 4.76 billion people, which corresponds to 59.4% of the population, use social media. In Turkey, there are 62.55 million active social media users, which accounts for 73.1% of the population, and a significant portion of internet users actively engage in social media (1). Due to the global pandemic, our government has implemented isolation regulations, resulting in students engaging in remote education from their homes and a significant portion of society relying on screens for extended periods of time for remote work. Media use encompasses all instances of screen exposure, including both interactive forms, such as gaming and social media, as well as passive forms like watching television (2, 3).

Due to features such as its continuous updateability, enabling multiple uses, and facilitating virtual sharing, social media is considered one of the preferred social interaction platforms among young people (4). With the development of mobile devices and operating systems, platforms like Instagram, Facebook, Twitter, and YouTube, widely used social networking sites, have made it easier for individuals to connect through content such as photos and videos, thus increasing the speed at which people influence each other (5). Social network usage has significantly increased, particularly during the COVID-19 pandemic, and reports suggest that young people in particular have been utilizing social media more frequently (6).

In recent times, there has been a noticeable increase in the use of the Internet to acquire information about nutrition, diet, and dietary supplements, and interest in these subjects continues to grow (7).

Social media offers advantages such as instant access to digital content from anywhere and the relatively inexpensive use of technologies such as smartphones and computers. These advantages have enabled university students, one of the most active users of social media, to more easily access nutrition-related content shared on social media platforms (8). Particularly in Turkiye, where the young population is abundant, social media can influence individuals’ food preferences and affect their choices (9). Social media gives people looking for nutrition information the chance to interact with a vast community and an extensive network. However, it also makes it challenging for them to determine whether the information being provided is trustworthy, accurate, and grounded in scientific principles (10). Increasing evidence suggests that young people are turning to social media more for health-related information in areas such as physical activity, diet/nutrition, and body image (11). As young people’s interest in the connection between social media and health grows, there is currently no advice on how to mitigate the hazards that could result (12). The freedom that comes with limitless sharing opportunities has led to a sharp rise in people exchanging information about diet, nutrition, health, and other topics of interest (13).

Therefore, the media literacy of the younger generation, who extensively utilize media tools and social media platforms, is crucial for laying the groundwork for future generations. Social media use (SMU) may serve students as a valuable tool for the exploration of new recipes, ingredients, and related nutritional information, thus enabling students to improve their eating behavior and diet quality. On the other hand, evidence linking SMU to negative eating thoughts, feelings, and behaviors suggests that the thought of excessive SMU has a negative impact on dietary quality. Therefore, this research was conducted to determine the association between university students’ SMU and diet quality.

## 2 Methods

### 2.1 Research design

Study data were collected between November 2021 and June 2022. The research was conducted on 1,241 university students who were randomly selected and participated in the research voluntarily. A simple random sampling method was used to keep the selection rate of all participants equal. Among the students interviewed, 14 people (8 female and 6 male) were not included in the study because they did not agree to participate. The sample consisted of 801 female students and 440 male students, all between the ages of 19 and 30. These students were enrolled in three universities located in Ankara, the capital city of Turkiye. The study consisted of students who were within the specified age range, studying in the universities where the research was carried out, without any chronic illnesses, and actively utilizing at least one social media account. Students who did not meet these criteria were not included in the study. The students included in the sample had not studied at another university before.

Because SMU was assessed dichotomously (rarely vs. frequently), we planned two-group comparisons. The required sample size was calculated using G*Power software for a two-tailed independent-samples t-test. Assuming a small-to-medium effect size, an alpha error probability of 0.05, and a desired power (1 − *β*) of 0.95, the minimum total sample size required was 820 participants. The final study sample (*n* = 1,241; 700 and 541 participants in the two groups, respectively) exceeded this requirement, confirming that the study achieved sufficient statistical power. The effect size was chosen according to Cohen’s conventional benchmarks, representing a small-to-medium difference between two independent groups. To enhance the representativeness and reliability of the results, the study included as many participants as possible. Consequently, the final sample adequately represented the target population of university students enrolled in the participating institutions. An attempt was made to increase the sample size as much as possible to exceed this number. As a result, it was determined that the sample size reached 1% of the general population in Ankara.

The study excluded individuals who did not fill out the survey, declined to provide their body weight and height information, or had a BMI below 18.5 kg/m^2^. The study was carried out with the approval of the Gazi University Ethics Committee, as indicated by approval number 2021-1064. Furthermore, the study was carried out in compliance with the principles outlined in the Helsinki Declaration. All participants provided written informed consent. Prior to the questionnaire, participants were asked to sign a written consent form.

In the study, researchers administered a questionnaire face-to-face, encompassing socio-demographic information, health and dietary habits, questions regarding SMU, and a 24-h retrospective dietary recall. Additionally, individuals’ Body Mass Index (BMI) was calculated using self-reported body weight and height measurements, applying the equation “weight/(height)^2^ (kg/m^2^),” and BMI classification was conducted using the cut-off values defined by the World Health Organization (14).

### 2.2 Social media usage

Participants were asked about the nutrition-related subjects they engage with on social media and the specific social media accounts they follow for these topics. The frequency of people’s social media usage was classified as “rarely” for those who accessed their social media accounts at least once a day and “frequently” for those who used social media more than once a day. This part, used to determine social media usage, was prepared by researchers. This part of the questionnaire was applied to a group of 10 people before the study, and it was observed whether there were any problems. After this pre-test, necessary arrangements were made. The survey responses from this pre-test group were not included in the study. The questionnaire also included categorical items identifying the sources of nutrition-related information followed on social media (e.g., Instagram, YouTube, Facebook). These items were descriptive and aimed to determine where participants obtained nutrition-related content.

Rationale for SM measure: The study focused on overall exposure frequency to social media rather than multidimensional engagement constructs. A brief, researcher-developed classification (rare vs. frequent daily access) was selected to ensure feasibility in a large sample, reduce respondent burden, and align the exposure with dietary outcomes (24-h recall, NAR/MAR). No nutrition-specific, validated Turkish instrument suitable for this population was available during planning; therefore, the item set was pilot-tested for clarity prior to fieldwork.

### 2.3 Assessment of dietary intake and quality

Food consumption of individuals was documented using a 24-h retrospective recall method. Since this method is more straightforward to use and aims to recollect information over a shorter time period, it is believed to produce more accurate findings. The BeBiS (Nutrition Information System) (version 8.2) was utilized to examine energy, macronutrients, and micronutrients. The Nutrient Adequacy Ratio (NAR) scores were determined by comparing the amount of food consumed by individuals on daily with the recommended levels of nutrients specified by the Dietary Reference Intake (DRI) for different age and gender groups (15). The study estimated the NAR scores for nine nutrients, including protein, carbohydrate, fiber, calcium, potassium, vitamin A, B12, folate, and vitamin C, as percentages. The Mean Adequacy Ratio (MAR) score was derived as a percentage by averaging the scores of the Nutrient Adequacy Ratios (NAR) measured for nine nutrients. Based on individuals’ MAR scores, diets with a score of ≤50 are categorized as “inadequate,” scores between 51 and 80 are classed as “needs improvement,” and scores above 80 are considered “good” (16).

### 2.4 Statistical analysis

The statistical data gathered from the investigation were examined using the SPSS 22.0 program. The normality of the data was assessed using the Kolmogorov–Smirnov Test. Statistical comparisons of normal distribution groups used the *t*-test. *T*-tests provided t (df) and effect size (Cohen’s *d*). Small, medium, and large effect sizes are indicated by Cohen’s d values, which are calculated using 0.2, 0.5, and 0.8 thresholds, respectively (17).

Parametric data in descriptive statistics is characterized by the use of mean (X̄) as the measure of central tendency and standard deviation (SD) as the measure of dispersion. The descriptive statistics of categorical variables involved the use of numbers (*n*) and percentages (%). Cross-tables and Chi-square analysis were employed to compare this data. The analysis of 2 × 2 categorical variables utilized *φ* statistics, while data with more than two categories were analyzed using Cramer’s V. Statistical significance was evaluated as *p* < 0.05.

## 3 Results

The general characteristics of the participants, including gender, age, BMI, supplement usage, skipping main meals, and the impact of social media sharing on personal dietary attitudes, are presented in Table 1 according to the participants’ social media usage status.

**Table 1:** General characteristics of individuals according to their SMU.

Characteristics	SMU		*t* (*df*), effect size^†^	*p*
	Rarely (*n*: 700)	Frequently (*n*: 541)		
	$\bar{x}$ ± SD	$\bar{x}$ ± SD		
Age (y)	21.1 ± 1.8	21.1 ± 2.3	−0.31 (1238), 0.01	0.759^#^
Body weight (kg)	64.3 ± 12.0	66.0 ± 12.5	−2.29 (1138.90), 0.14	**0.022** ^**#**^
Height (cm)	167.7 ± 8.5	169.1 ± 9.2	−2.78 (1235), 0.16	**0.006** ^**#**^
BMI (kg/m^2^)	22.7 ± 3.1	22.9 ± 3.0	−1.05 (1239), 0.06	0.293^#^

	*n* (%)	*n* (%)	*ꭓ*^2^ (*df*), φ/Cramer’s *V*	*p*
Gender**				
Male	227 (51.6)	213 (48.4)	6.428 (1), 0.072^§^	0.011*
Female	473 (59.0)	328 (41.0)		
BMI (kg/m^2^)				
Normal	569 (81.3)	483 (81.0)	0.088 (2), 0.008^¶^	0.957*
Overweight	109 (15.6)	87 (16.1)		
Obese	22 (3.1)	16 (3.0)		
Supplement use				
Yes	83 (11.9)	59 (10.9)	0.273 (1), 0.015^§^	0.602*
No	617 (88.1)	482 (89.1)		
Skipping main meals				
Yes	524 (74.9)	409 (75.6)	1.222 (2), 0.031^§^	0.543*
No	176 (25.1)	132 (24.4)		
Department classification				
Health related	341 (48.7)	222 (41.0)	7.260 (1), 0.076^§^	0.007*
Others	359 (51.3)	319 (58.9)		

SMU, social media use; BMI, body mass index. Percentages calculated by column. *p* < 0.05 (Significant results are shown in bold). ^#^Independent sample *t*-test. ^§^φ Statistics. ^¶^Cramer’s *V*. *Chi-square test. ^†^Cohen’s *d*, interpreted as small (0.2), medium (0.5), and large (0.8) effects. **Percentages were calculated based on gender.

The study encompassed a cohort of 1,241 university students, consisting of 700 individuals classified as rarely users of social media and 541 individuals classified as frequent users. The mean age of the included participants was 21.1 ± 1.8 years for rarely users and 21.1 ± 2.3 years for frequently users (*p* > 0.05). Body weight of the participants was 64.3 ± 12.0 and 66.0 ± 12.5 for rarely and frequently users, respectively, *t* (1138.90) = −2.29, Cohen’s *d* = 0.14 (small effect), *p* = 0.022. Accordingly, the body weight of those who frequently use social media was found to be significantly higher than that of those who rarely use it. Despite the importance of body weight, the BMI of the participants was 22.7 ± 3.1 kg/m^2^ for rarely users, 22.9 ± 3.0 for frequently users, *t* (1239) = −1.05, Cohen’s *d* = 0.06 (small effect), *p* = 0.293. Accordingly, it is seen that the frequency of social media use does not give a statistically significant result with BMI.

According to the study, 48.4% of males and 41.0% of females reported frequent use of social media, while 51.6% of males and 59.0% of females reported rare use. This indicates that frequent social media use was slightly more common among males than females (*ꭓ*^2^(1) = 6.428, Cramer’s *V* = 0.072, *p* = 0.011). When supplement use and main meal skipping were evaluated according to the frequency of SMU, no statistical significance was found (*p* > 0.05). Upon reviewing the departments in which students are studying, it was discovered that 48.7% of students who use social media rarely and 41.0% of students who use it frequently are enrolled in health-related departments, such as nursing, medicine, and nutrition and dietetics. In contrast, 51.3% of individuals who use social media infrequently and 58.9% of those who use it frequently are studying in departments that are not related to health, such as engineering, business, and others. (*ꭓ*^2^(1) = 7.260, Cramer’s *V* = 0.076, *p* = 0.007). In other words, it is seen that people with professions not related to health use social media more frequently, which is statistically significant.

The distribution of participants’ nutritional attitudes according to SMU is shown in Table 2. When investigating the impact of individuals’ social media shares on their personal nutrition attitudes, a statistically significant difference was observed in terms of nutritional knowledge obtained from social media and the implementation of detox programs through social media, as compared to their social media usage status (*p* < 0.05).

**Table 2:** Distribution of nutritional attitudes according to SMU.

Characteristics	SMU		*t* (*df*), effect size^†^	*p*
	Rarely (*n*: 700)	Frequently (*n*: 541)		
	$\bar{x}$ ± SD	$\bar{x}$ ± SD		
News on social media changes my attitude toward some foods				
Always	27 (3.9)	20 (3.7)	1.666 (3), 0.037^¶^	0.654*
Sometimes	381 (54.4)	313 (57.9)		
Rarely	207 (29.6)	144 (26.6)		
Never	85 (12.1)	64 (11.8)		
I get nutritional knowledge from social media				
Always	41 (5.9)	39 (7.2)	9.299 (3), 0.087^¶^	0.026*
Sometimes	271 (38.7)	230 (42.5)		
Rarely	239 (34.1)	189 (34.9)		
Never	149 (21.3)	83 (15.3)		
I follow the weight loss diets on social media				
Always	9 (1.3)	6 (1.1)	6.529 (3), 0.73^¶^	0.089*
Sometimes	64 (9.1)	69 (12.8)		
Rarely	128 (18.3)	113 (20.9)		
Never	499 (71.3)	353 (65.2)		
I believe that weight loss products advertised on social media are reliable				
Always	9 (1.3)	7 (1.3)	1.695 (3), 0.037^¶^	0.638*
Sometimes	31 (4.4)	19 (3.5)		
Rarely	78 (11.1)	71 (13.1)		
Never	582 (83.1)	444 (82.1)		
I purchase dietary supplements that are advertised on social media				
Always	9 (1.3)	4 (0.7)	2.129 (3), 0.041^¶^	0.546*
Sometimes	26 (3.7)	26 (4.8)		
Rarely	67 (9.6)	57 (10.5)		
Never	598 (85.4)	454 (83.9)		
I believe that online diets are reliable				
Always	16 (2.3)	13 (2.4)	1.422 (3), 0.034^¶^	0.700*
Sometimes	123 (17.6)	92 (17.0)		
Rarely	184 (26.3)	157 (29.0)		
Never	377 (53.9)	279 (51.6)		
I apply detox programs on social media				
Always	7 (1.0)	13 (2.4)	14.959 (3), 0.11^¶^	0.002*
Sometimes	61 (8.7)	79 (14.6)		
Rarely	143 (20.4)	104 (19.2)		
Never	489 (69.9)	345 (63.8)		

SMU, social media use. Percentages calculated by column. *p* < 0.05 (Significant results are shown in bold). ^#^Independent sample *t*-test. ^§^φ Statistics. ^¶^Cramer’s *V*. *Chi-square test. ^†^Cohen’s *d*, interpreted as small (0.2), medium (0.5), and large (0.8) effects.

Sources of nutrition-related accounts followed by students according to their usage status on social media are shown in Table 3. Regardless of the students’ frequency of SMU, Instagram is the leading nutrition-related account they follow on social media, followed by YouTube. When the sources that follow healthy nutrition recipes are evaluated according to social media usage status, a statistical significance was found (*p* < 0.05).

**Table 3:** Sources of nutrition-related accounts followed according to their SMU.

Nutrition-related accounts	SMU		*p*
	Rarely	Frequently	
General information about nutrition			
Instagram	228 (51.4)	216 (48.6)	0.266
Facebook	13 (65.0)	7 (35.0)	
Pinterest	2 (66.7)	1 (33.3)	
Twitter	4 (28.6)	10 (71.4)	
Tumblr	–	1 (100.0)	
YouTube	44 (52.4)	40 (47.6)	
Healthy eating recipes			
Instagram	206 (50.0)	206 (50.0)	**0.047**
Facebook	11 (55.0)	9 (45.0)	
Pinterest	3 (75.0)	1 (25.0)	
Twitter	–	7 (100.0)	
Tumblr	3 (75.0)	1 (25.0)	
YouTube	78 (58.2)	56 (41.8)	
Online diet			
Instagram	109 (51.9)	101 (48.1)	0.231
Facebook	7 (58.3)	5 (41.7)	
Pinterest	3 (75.0)	1 (25.0)	
Twitter	1 (14.3)	6 (85.7)	
Tumblr	1 (50.0)	1 (50.0)	
YouTube	15 (57.7)	11 (42.3)	
Weight loss products			
Instagram	85 (50.6)	83 (49.4)	0.070
Facebook	6 (75.0)	2 (25.0)	
Pinterest	2 (66.7)	1 (33.3)	
Twitter	–	4 (100.0)	
Tumblr	–	2 (100.0)	
YouTube	17 (56.7)	13 (43.3)	

SMU, Social media use. There are individuals who do not follow nutrition-related accounts. Therefore, percentages were calculated based on the number of individuals responding. *p* < 0.05 (Significant results are shown in bold).

The participants’ SMU was classified according to the department, and the comparison of energy intake, NAR, MAR scores, and MAR classifications is presented in Table 3. Based on this information, it was observed that students in health-related departments had a considerably reduced energy intake compared to students in other departments (*p* < 0.05). In contrast, students enrolled in health-related departments exhibited elevated consumption of fiber and vitamin A in comparison to students in other departments. Nevertheless, their intake of folate was seen to be decreased (*p* < 0.05).

When students’ SMU was evaluated according to their departments, whether they were in a health-related department or not, the energy, protein, and carbohydrate intakes of those who used social media rarely were not found to be significantly different from those who used it frequently (*p* > 0.05). Calcium and potassium levels of those studying in the health department who use social media frequently were found to be significantly higher than those who use social media rarely (*p* < 0.05). There was no significant difference found in the MAR scores and MAR classifications among participants based on their academic departments and social media usage classifications (*p* > 0.05) (Table 4).

**Table 4:** Comparison of individuals’ energy intake, NAR, MAR scores, and MAR classifications according to their SMU.

Variables	Health related department (*n*: 563)			Non-health related department (*n*: 678)			p*
	All (*n*: 563)	SMU		All (*n*: 678)	SMU		
		Rarely (*n*: 341)	Frequently (*n*: 222)		Rarely (*n*: 359)	Frequently (319)	
	$\bar{x}$ ± SD	$\bar{x}$ ± SD	$\bar{x}$ ± SD	$\bar{x}$ ± SD	$\bar{x}$ ± SD	$\bar{x}$ ± SD	
Dietary intake							
Energy	1680.9 ± 515.5	1669.7 ± 492.0	1698.0 ± 550.3	1746.1 ± 605.4	1738.7 ± 597.5	1754.5 ± 615.1	**0.040**
		0.05^†^, *p* = 0.525			0.03^†^, *p* = 0.735		
NAR scores							
Protein	95.0 ± 11.9	94.7 ± 12.2	95.4 ± 11.5	93.7 ± 13.9	93.3 ± 14.4	94.1 ± 13.3	0.084
		0.06^†^, *p* = 0.450			0.06^†^, *p* = 0.434		
Carbohydrate	89.1 ± 19.2	89.7 ± 18.7	88.3 ± 20.0	88.4 ± 19.5	87.1 ± 20.5	89.9 ± 18.3	0.511
		0.07^†^, *p* = 0.382			0.14^†^, *p* = 0.063		
Fiber	62.5 ± 23.6	62.2 ± 23.1	62.9 ± 24.2	59.5 ± 24.1	60.6 ± 25.3	58.2 ± 22.7	**0.028**
		0.03^†^, *p* = 0.748			0.10^†^, *p* = 0.190		
Calcium	56.3 ± 24.3	54.6 ± 23.9	58.9 ± 24.6	57.9 ± 25.4	57.9 ± 26.0	58.0 ± 24.8	0.250
		0.18^†^, ***p* = 0.042**			0.00^†^, *p* = 0.987		
Potassium	43.7 ± 16.5	42.3 ± 14.8	45.7 ± 18.6	45.5 ± 18.1	45.7 ± 18.2	45.2 ± 18.0	0.062
		0.20^†^, ***p* = 0.024**			0.03^†^, *p* = 0.726		
Vitamin A	74.8 ± 26.7	75.8 ± 26.8	73.3 ± 26.5	71.4 ± 27.7	71.4 ± 28.5	71.4 ± 26.9	**0.030**
		0.09^†^, *p* = 0.272			0.00^†^, *p* = 0.999		
Vitamin B_12_	87.9 ± 23.4	88.5 ± 23.5	87.1 ± 23.3	89.2 ± 22.3	87.9 ± 24.1	90.7 ± 19.9	0.320
		0.06^†^, *p* = 0.499			0.13^†^, *p* = 0.100		
Folate	54.5 ± 26.8	54.1 ± 26.2	55.0 ± 27.9	58.9 ± 24.8	59.1 ± 25.1	58.7 ± 24.5	**0.003**
		0.03^†^, *p* = 0.695			0.02^†^, *p* = 0.858		
Vitamin C	74.4 ± 31.1	74.9 ± 29.8	73.6 ± 33.1	70.8 ± 32.6	71.2 ± 33.2	70.4 ± 31.9	0.051
		0.04^†^, *p* = 0.634			0.02^†^, *p* = 0.761		
MAR [ $\bar{x}$ ± SD]	71.8 ± 14.3	71.5 ± 14.0	72.1 ± 14.8	72.2 ± 14.8	72.0 ± 15.3	72.4 ± 14.2	0.616
		0.04^†^, *p* = 0.600			0.03^†^, *p* = 0.753		

MAR classification	*n* (%)	*n* (%)	*n* (%)	*n* (%)	*n* (%)	*n* (%)	*p*
Inadequate	47 (8.3)	28 (8.2)	19 (8.6)	59 (8.7)	30 (8.4)	29 (9.1)	0.960
Needs improvement	335 (59.5)	221 (61.9)	124 (55.8)	405 (59.7)	211 (58.8)	194 (60.8)	
Good	181 (32.2)	102 (29.9)	79 (35.6)	214 (31.6)	118 (32.8)	96 (30.1)	
		*p* = 0.335			*p* = 0.728		

SMU, Social media use. Percentages are calculated by column. NAR, Nutrient adequacy ratio; MAR, Mean adequacy ratio. *p* < 0.05 (Significant results are shown in bold). p* for the participants’ was classified according to the department. ^†^Cohen’s *d*, interpreted as small (0.2), medium (0.5), and large (0.8) effects.

## 4 Discussion

The study aimed to determine the dietary topics followed by university students based on their frequency of social media usage and the social media platforms they follow, and to evaluate individuals’ behaviors toward these posts and their dietary qualities. Regarding the BMI distribution, supplement use, and main meal skipping among the subjects, there was no difference detected based on their social media usage status. The acquisition of dietary information from social media did not differ among groups according to the frequency of social media following. Furthermore, participants were queried about their trust in weight loss products advertised on social media and whether they purchased nutritional products presented on these platforms, with the majority indicating distrust and non-purchase. Additionally, more than half of the participants stated that they did not implement detox programs found on social media. Finally, there was no significant difference in the NAR scores, MAR scores, and MAR classification of nine nutrients among participants based on their social media usage status. In light of this information, it was concluded that university students participating in the research with at least one or more social media accesses per day actively engaged with nutrition-related content; however, the information presented on social media did not negatively influence individuals’ dietary attitudes and qualities.

Social media has become an integral part of modern society, serving as a significant communication tool that influences people’s ways of connecting, accessing information, and sharing opinions across all segments of society. Particularly, university students are among the most active users of social media, with over 90% of young individuals reported to have at least one social media account (18, 19). Among the participants in this study, 89.3% (48.4% male, 40.9% female) were found to use social media multiple times a day (data not shown). This widespread use of social media among young people provides opportunities for accessing information more rapidly and efficiently, thereby increasing interest in the potential health outcomes of social media influence within this group (20). Platforms such as Facebook, YouTube, or Instagram facilitate the sharing of various information related to nutrition, physical activity, and health, not only by experts in the field but also by the general public, including non-healthcare professionals and influencers (21, 22). Nutrition content shared on social media platforms encompasses a wide range of information, from detox products to recipes, healthy lifestyle tips, and diet trends. While some content provides information about healthy and balanced nutrition, it may also popularize unhealthy eating patterns (23). In this study, 38.7% of young individuals who infrequently use social media and 42.5% of frequent users indicated obtaining nutrition-related information from social media (*p* > 0.05) (Table 1). The comprehensive nature of information available on social media allows young people to access various nutrition recommendations easily. However, it is important to question who provides these recommendations and to evaluate the scientific basis of the information.

It has been noted that social media usage can increase body dissatisfaction and lead individuals to adopt thinness ideals, which may result in risky eating behaviors (24). Studies have shown a positive relationship between the frequency of Facebook use and depression, anxiety, body dissatisfaction, and body shame (25, 26). Not only in females but also in both genders, individuals with high Facebook engagement (passive/active usage) have been reported to experience increased objectified body consciousness, a sign of body shame (27). As social media users are exposed to non-scientifically based nutrition and health advice more frequently, their eating behaviors may change, leading to an increased desire to consume unhealthy foods (28). A study indicated that maladaptive Facebook use increased bulimic symptoms and binge eating episodes (29). Furthermore, strong associations between SMU and irregular eating patterns have been observed in young adults, with an increase in time spent on Instagram and Snapchat paralleling an increase in irregular eating behaviors (30). Another study found that while Facebook use among college students reduced junk food consumption, it did not significantly improve healthy eating and physical activity (31). In this study, although no difference was found between groups in terms of the effect of news appearing on these platforms on attitudes toward foods (*p* > 0.05) when evaluated based on the frequency of social media usage, 54.4% of infrequent users and 57.9% of frequent users stated that their attitude toward foods sometimes changed (*p* > 0.05) (Table 1). This situation may indicate that young individuals approach nutrition-related information on social media more cautiously and rationally.

Social media also presents significant opportunities to positively impact public health by disseminating information that can prevent obesity. These platforms can be used to spread evidence-based information about habits and behaviors that promote health. Recent evidence supports the use of social media platforms to increase interventions aimed at promoting and improving health (23). In this study, Instagram ranks among the top sites followed by university students for nutrition-related accounts and the participants’ approach to online diet programs was found, as shown in Table 1. Social media-based nutrition education can provide students with a tool for widespread and timely access to facilitate healthy eating behaviors (32). Social media is a potentially attractive way to support adolescents and young adults in maintaining and learning about healthy eating habits (33). A Twitter-based nutrition intervention for university students focusing on nutrition knowledge, dietary practices, self-efficacy, and social support has been reported to increase personal awareness and attempt to instill healthy eating habits (34).

However, the results of this study show that SMU does not affect NAR and MAR scores. Another study of young adults in New Zealand found that although there was a relationship between social media and body image, the effect on eating behaviors and diet quality was weak (35). A review of different studies on the subject over the last 10 years found that social media use generally has adverse effects on diet quality and encourages irregular eating patterns (36). According to these results, the relationship between social media and diet quality may be affected by various factors (such as cultural differences).

In conclusion, due to the rapid increase in social media usage in recent years, nutrition and health professionals, as well as government and non-governmental health organizations, are striving to utilize social media to strengthen healthy food choices and nutrition-related behaviors among young adults (19). University students can find content on social media that promotes healthy lifestyles. They can use these platforms to increase health awareness and develop healthy eating habits. However, with increased exposure to social media, negative body image and irregular eating behaviors can be observed among young individuals. Therefore, young people need to manage their social media usage consciously. The reliability of information regarding healthy eating should be questioned, and the scientific basis of every trend or diet should be researched. Additionally, health professionals can guide students on SMU.

There were several limitations and strengths to the present study. The strengths of the study lie in its comprehensive approach to investigating the impact of social media on university students’ perceptions of nutritional quality. The inclusion of a large sample size and the utilization of diverse methodologies, such as surveys and dietary recall records, enhances the robustness of the findings. Additionally, the study’s focus on various aspects of SMU, including frequency and preferred platforms, provides valuable insights into students’ behavior. Notwithstanding these advantages, it is important to consider the following limitations when interpreting this work. The SMU can serve as a valuable tool for students about nutrition, but the SMU was used in this study as a researcher-designed survey, not a scale, and therefore may not reflect accurate results. Self-reported data on age, weight, and height could introduce biases, and reliance on participants’ memory for dietary recall may affect the accuracy of nutritional assessment. The grouping made at the beginning of the study regarding the frequency of social media use is one of the factors that may limit the participants. Since the students’ fields of expertise are not questioned, the answers of those studying in a nutrition-related field may be influenced by their academic knowledge level. Furthermore, the study’s cross-sectional design limits the ability to establish causal relationships between social media use and nutritional habits. Additionally, the study’s findings may not be generalizable to populations outside of university settings. Also, the reliability of NAR/MAR ratings may be impacted since a single 24-h recall would not accurately reflect habitual intake.

Young adults frequently utilize social media platforms as a means for sharing information related to nutrition. The impact of social media usage on nutrition among university students is complex. Although social media can encourage unhealthy eating patterns, it also presents avenues for implementing public health campaigns that support healthy food choices. Students should utilize social media platforms with mindfulness in order to obtain accurate, reliable information and develop healthy eating habits. Furthermore, educational institutions and nutritionists need to enhance awareness and offer comprehensive advice to university students regarding the impacts of social media. Future studies should prioritize the exploration of optimal strategies for captivating young people through social media, enhancing the efficacy of social media in assisting young adults, and facilitating social and peer support to promote healthier choices.

## Data Availability

The raw data supporting the conclusions of this article will be made available by the authors, without undue reservation.
